# Advances in Therapeutic Cholangioscopy

**DOI:** 10.1155/2016/5249152

**Published:** 2016-06-15

**Authors:** Tomazo Antonio Prince Franzini, Renata Nobre Moura, Eduardo Guimarães Hourneaux de Moura

**Affiliations:** Department of Gastroenterology, Endoscopy Unit, University of Sao Paulo, Avenida Dr. Eneas de Carvalho Aguiar 255, 6° Andar, Bloco 3, 05403-000 Sao Paulo, SP, Brazil

## Abstract

Nowadays, cholangioscopy is an established modality in diagnostic and treatment of pancreaticobiliary diseases. The more widespread use and the recent development of new technologies and accessories had renewed the interest of endoscopic visualization of the biliary tract, increasing the range of indications and therapeutic procedures, such as diagnostic of indeterminate biliary strictures, lithotripsy of difficult bile duct stones, ablative techniques for intraductal malignancies, removal of foreign bodies and gallbladder drainage. These endoscopic interventions will probably be the last frontier in the near future. This paper presents the new advances in therapeutic cholangioscopy, focusing on the current clinical applications and on research areas.

## 1. Introduction

In recent decades, endoscopic retrograde cholangiopancreatography (ERCP) had been the primary tool in the endoscopic treatment of biliary tract diseases, with success rates above 90% [1–3]. Nevertheless, some situations remain challenging, with difficult diagnosis and treatment, as indeterminate biliary strictures and stones of difficult removal.

In this context, cholangioscopy was introduced in order to improve diagnostic and therapy of biliary diseases, allowing direct visualization of the biliary system and also performance of therapeutic interventions [4].

Endoscopic evaluation of the biliary tree is not a recent procedure, as some may believe. The first optical choledocoscope was developed in 1941 and the peroral approach in 1976, initially through a prototype that was thin enough to be inserted through the working channel of the duodenoscope [5, 6]. This system was known as “mother-baby scope,” requiring two endoscopists for its management. This first prototype had a fiber-optic camera, low quality, and neither working channels nor irrigation and was without tip deflection. Its use remained limited, mainly due to the high cost, fragility, and requirement of two experienced endoscopists. Despite these limitations, it was possible to prove that the peroral cholangioscopy was feasible [7–9].

From the mid-80s, the second generation of cholangioscopes was developed with larger diameters, tip deflections, and working channels, allowing the introduction of instruments and irrigation [6]. Also, with the advancement of technology, there was improvement of the image allowing proper evaluation of the biliary tract mucosa and lumen.

**Figure 1:**
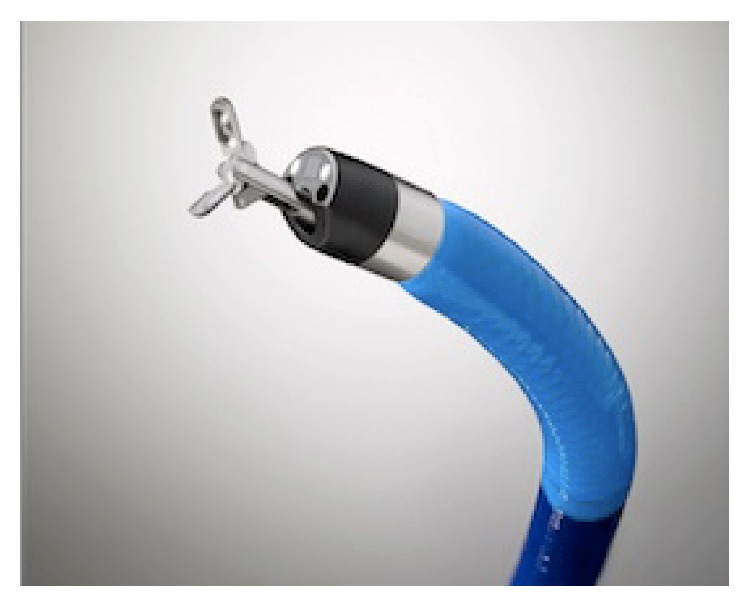
SpyGlass DS: cholangioscopy single-operator platform.

In 2007, the first cholangioscopy platform was introduced with a unit of single-operator (SpyGlass®), making the procedure more feasible and effective, enabling accurate biopsies and lithotripsy under direct visualization. Consequently, for its numerous advantages, the use of SpyGlass gained popularity, with great advantages in everyday practice [2]. In 2014, the second generation was introduced, with higher quality image (SpyGlass DS®), and also important improvements as ergonomics, stability, accessory exposure, and larger working channel (Figure 1).

Single-operator system with “ultraslim” endoscopes with an external diameter ranging from 5 to 6 mm can also be used. Because of its diameter, the presence of dilated biliary duct and previously sphincterotomy is necessary. The major advantage is the superior digital image quality [2, 3], especially desirable in diagnostic procedures.

The recent development of new technologies, including high-definition images and the incorporation of optical chromoendoscopy (NBI), has renewed interest in endoscopic visualization of the biliary tree and led to more widespread use of cholangioscopy [4, 10–15]. Cholangioscopy modalities are summarized in Table 1.

It is a fact that most of cholangioscopy indications are to evaluate indeterminate biliary strictures. In the present paper, we have focused on the advances of therapeutic cholangioscopy, highlighting the large potential of this issue in our clinical practice. Diagnostic applications are not on the scope of this issue. Currently, the established indication of cholangioscopy in therapeutic field is to treat difficult biliary stones, when associated with electrohydraulic lithotripsy (EHL) or laser lithotripsy (LL). Nevertheless, the indications continued to expand and several applications have been described, such as treatment of biliary strictures, lithotripsy of pancreatic duct stones, tumor ablation, gallbladder and biliary drainage, guidewire placement, foreign body removal, and the diagnosis and treatment of hemobilia, as discussed below.

## 2. Lithotripsy for Difficult Biliary Stones

Approximately 10–15% of stones cannot be extracted by ERCP conventional methods under certain circumstances, needing additional or other therapeutic modalities [15, 16]. Furthermore, it has been shown that previous ERCPs failed to correctly identify choledocholithiasis in 8%–16% of cases [14].

The definition of difficult bile duct stones is complex and involves many factors such as size (usually stones larger than 15 mm), disproportion of the stone with the distal common bile duct, postoperative anatomical changes, stenosis, presence of multiple and barrel-shaped stones, and inaccessible locations (intrahepatic stones, the Mirizzi syndrome) [11, 13, 15].

**Figure 2:**
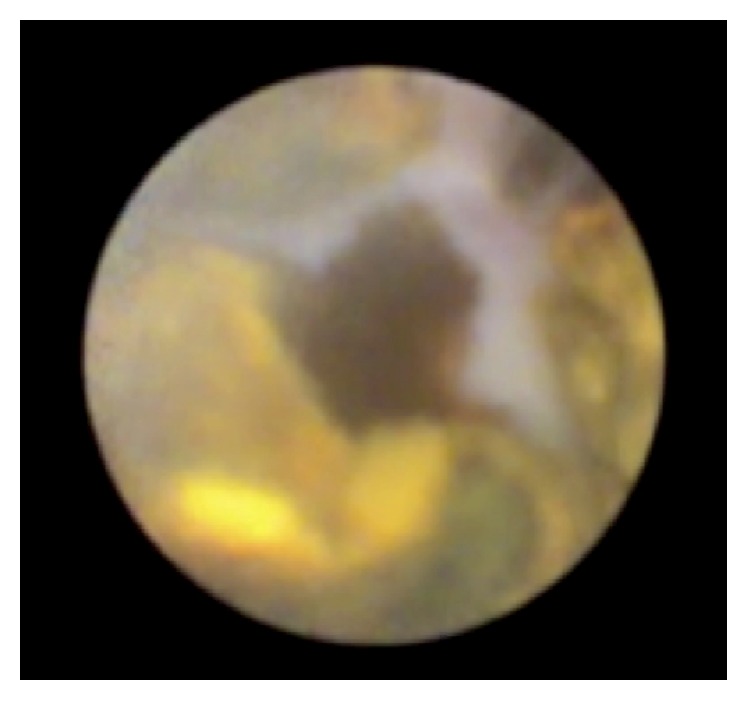
Broken bile duct large stone after EHL session.

Lithotripsy guided by cholangioscopy allows stone fragmentation through electrohydraulic waves (EHL) (Figure 2) or laser (LL), facilitating the subsequent removal with conventional accessories. Several studies report success rates of 80–90% and these results are frequently achieved in just one session [2, 9, 11–14]. Thus, lithotripsy under direct visualization is safer because it helps prevent bile duct injury and reduces the need for mechanical lithotripsy [4, 15].

EHL is performed through a 1.9 Fr nitinol fiber containing two electrodes in its tip. High amplitude hydraulic pressure waves are created, requiring immersion in saline solution. A generator produces a series of electrical pulses of high voltage at a frequency of 1 to 20 seconds, with a power of 50 to 100 watts.

LL is performed using a pulse holmium YAG or aluminum transmitted through a flexible quartz fiber. The application of repeated pulses of energy leads to accumulation of gaseous ions and free electrons, inducing a wave of mechanical shock and causing stone fragmentation. Irrigation is necessary to allow laser propagation and to ensure adequate clearance from the duct during the procedure.

The effectiveness of the electrohydraulic and laser lithotripsy is similar in terms of stone fragmentation rates, but LL seems to be more expensive and requires more time [4, 15].

In case of intrahepatic stones, the thinner LL probe is generally preferred to the EHL probe, whereas the EHL is the most widely used technique, particularly with the SpyGlass system, because of the dedicated irrigation channel providing the flowing water that is required to perform the EHL [14]. Percutaneous transhepatic cholangioscopy- (PTCS-) EHL/laser lithotripsy is probably the only alternative to surgery for removal of intrahepatic stones [15, 17, 18].

Regarding the Mirizzi syndrome, the conventional management has been surgical and endoscopic treatment is still controversial, except to relieve a bile duct obstruction, with limited data regarding the effectiveness or complication rate of this approach [19–21]. Binmoeller et al. [22] demonstrated 100% success when treating 14 patients with Mirizzi's syndrome and Tsuyuguchi et al. [21] successfully treated 23 of 25 patients (92%), concluding that endoscopic treatment of patients with the Mirizzi syndrome is effective and less invasive compared with surgery in those with type II syndrome. In patients with type I, the stones may not be accessible to the cholangioscope, and surgery may be preferable.

## 3. Ablation Techniques

The ablative therapies for intraductal cancer guided by cholangioscopy are increasingly being applied and aim to improve cholestasis, survival, and quality of life [15]. These techniques include various forms and can be performed directly (e.g., brachytherapy and radiofrequency ablation) or indirectly (e.g., photodynamic therapy).

### 3.1. Photodynamic Therapy (PDT)

PDT has become an ascending mode for the treatment of unresectable cholangiocarcinoma and involves intravenous administration of a photosensitizer which is accumulated preferentially in tumor cells, followed by exposure of the tissue to the photocuring light, by generating cytotoxic reaction and subsequently ischemia, necrosis, and apoptosis of tumor cells. In many studies, patients undergoing PDT showed an increased survival rate compared with conventional stenting alone [23, 24]. Cholangioscopy may be useful for determining the extent of the spread of bile duct tumors and the appropriate location of the diffuser for light activation as well as for evaluating the clinical response to PDT.

Ortner et al. [24] performed a randomized control trial comparing stenting + PDT with stenting alone in 39 patients with histologically confirmed cholangiocarcinoma. PDT resulted in prolongation of survival (*P* < 0.0001). It also improved biliary drainage and quality of life. This study was terminated prematurely because PDT proved to be so superior to simple stenting treatment that further randomization was deemed unethical. Other studies also proved the advantages of PDT [25].

### 3.2. Radiofrequency Ablation (RFA)

RFA is the most promising endoscopic ablative technique nowadays due to its potential benefits, including reduced mortality and morbidity [23]. It is performed through catheters that induce thermal damage to the tissue by electromagnetic energy. Direct cholangioscopy can be useful in confirming a successful response to therapy.

Several authors [23, 26] described the feasibility and effectiveness of this technique; however, more randomized controlled trials are needed to compare its benefit against other treatments.

### 3.3. Brachytherapy

Intraductal brachytherapy (IB) is performed using a catheter positioned directly into the biliary stricture area, to apply iridium-192 isotopes. Radiation doses may vary from 10.4 to 20 Gy. It has the advantage of affecting only the desired location and a small area around, preventing tumor growth and avoiding unnecessary irradiation. It can be performed either endoscopically or percutaneously [23].

The effectiveness of this technique remains controversial in literature. Montemaggi et al. [27] described 12 patients submitted to intraluminal brachytherapy (eight on the bile duct and four on pancreatic duct). The results suggested that the addition of IB after biliary drainage prolongs survival. However, complications as cholangitis and gastrointestinal toxicities occurred in nine patients. Deodato et al. [28] evaluated long-term effects of IB, with clinical response rate of 28.6%, complete response in 9%, and median survival of 23 months. In conclusion, the role of IB in biliary cancer may be further analyzed in larger clinical trials.

## 4. Foreign Body Removal

Cholangioscopy-guided foreign body removal has been described in some case series.

Hasan et al. [29], using the new digital SpyGlass cholangioscope, performed direct endoscopic evaluation of a benign biliary stricture and identified a staple protruding through the biliary mucosa, which could have been a nidus for stricture formation. The staple was then removed by using SpyBite® biopsy forceps.

Basket impaction of a bile duct stone is a well-known problem occurring during endoscopic transpapillary lithotripsy. Generally, it is resolved by a transoral endotripter. However, even if the endotripter is used, sometimes it failed when the wires break because of the hardness of the stone. Wong et al. and Tsuchiya et al. [30, 31] described a successful removal of basket-impacted stone by use of transpapillary cholangioscopic electrohydraulic lithotripsy (EHL) and laser.

Cholangioscopy can be a useful tool to remove occluded or migrated biliary stents that cannot be removed with conventional techniques. Sanaka et al. and Sejpal et al. [32, 33] performed a retrieval of migrated biliary stent with direct peroral cholangioscopy, one by grasping with a thin snare and the other by cannulating with a guidewire and a stent retriever. Ikeura et al. [34] described a reintervention for an occluded metal stent under the guidance of peroral direct cholangioscopy by using an ultraslim enteroscope.

## 5. Guidewire Placement

**Figure 3:**
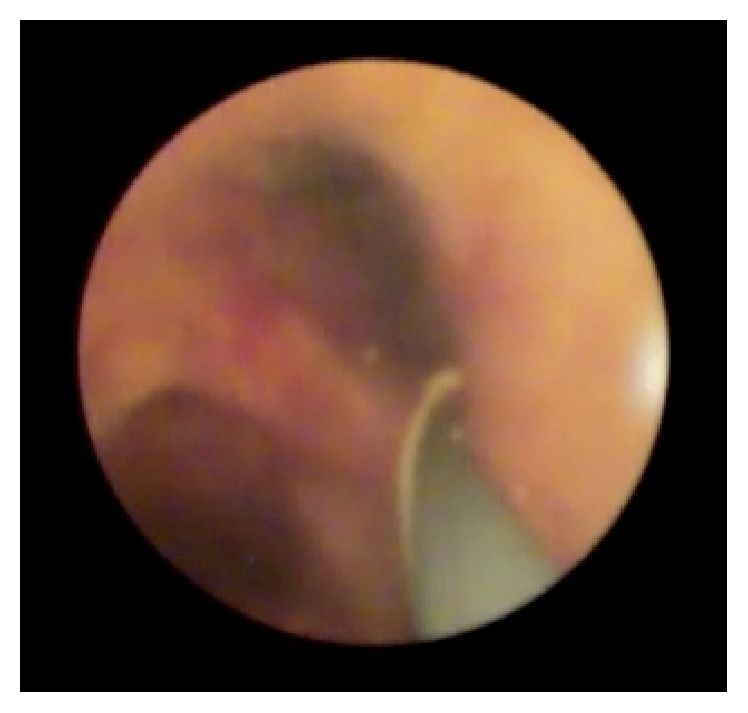
Direct view cholangioscopy enabling the adequate placement of guidewire through a biliary stricture.

Occasionally, guidewire placement can be a challenge, requiring more invasive procedures, such as percutaneous access or surgery. Using a cholangioscope, under direct visualization, the guidewire can be easily manipulated and placed in the desired location [35] (Figure 3).

## 6. Gallbladder Drainage

The gold standard treatment for acute cholecystitis is surgery. Nevertheless, some patients are not amenable due to significant comorbidities. In this case, percutaneous cholecystostomy is an alternative to surgery. Although a simple procedure, there are several complications, rating from 9 to 27% and including hemobilia, hematoma, and bile leak. When this technique is contraindicated or anatomically inaccessible, endoscopic-guided drainage can be used [36–38].

Cholangioscopy has significant advantages over ERCP in allowing direct visualization of the bile duct and obtaining targeted cystic duct cannulation. Itoi et al. [39] published a systematic review that revealed that endoscopic gallbladder stenting had a technical success rate of 96% and a clinical success rate of 88% which compared favorably with percutaneous transhepatic gallbladder drainage (98% and 90%, resp.). More investigations that compare cholangioscopy-assisted procedures and those without cholangioscopy are needed to evaluate the efficacy of this technique.

Shin et al. [36] reported 8 cases of SpyGlass-assisted gallbladder drainage, with a technical and clinical success rate of 88% and 75%, respectively. Complications such as pancreatitis, bleeding, and perforation did not occur in any patient.

## 7. Hemostasia

There are few cases reporting cholangioscopy diagnostic and therapeutic of bleeding lesions in the biliary mucosa [40–42]. Komaki et al. [40] reported a case of argon plasma coagulation under direct peroral cholangioscopy in a patient with hereditary hemorrhagic telangiectasia and repeated hemobilia.

## 8. Postliver Transplant Biliary Stricture

Cholangioscopy has been very useful in the evaluation and treatment of biliary complications after liver surgery. Direct visualization of the bile ducts may be a useful adjunct to endoscopic retrograde cholangiopancreatography (ERCP) for the evaluation of biliary strictures [43, 44]. Cholangioscopy increases the ability to evaluate mucosal changes and presence of fibrosis and provides direct intraductal therapies.

**Figure 4:**
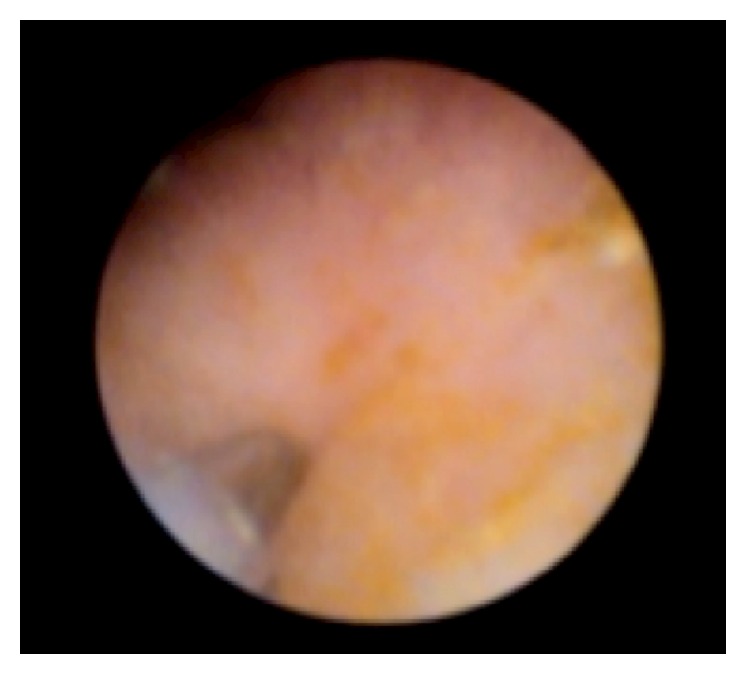
Cholangioscopy-guided steroid injection.

The safety and feasibility of single-operator cholangioscopy-guided steroids injection has been demonstrated by Franzini et al. [45] in a patient with refractory anastomotic biliary stricture after liver transplant. The patient underwent two sessions of cholangioscopy-guided steroid injection immediately after biliary balloon dilation, with 40* *mg of triamcinolone acetate injected per session (Figure 4). It was the first report of a benign biliary stricture (BBS) treated by extreme balloon dilation combined with cholangioscopy-guided steroid injection. Randomized controlled trials could confirm if this technique has the potential to become a standard treatment for refractory BBS.

Severe anastomotic stricture after living donor transplant is a challenge to endoscopic treatment, mainly due to the inability to advance the guidewire through the stenotic area. In these cases, cholangioscopy commonly enables successful guidewire placement as described in report cases [46–48].

Another interesting field of application cholangioscopy is the evaluation and treatment of biliary cast syndrome, a condition usually associated with biliary strictures and hepatic ischemia after liver transplant. Navaneethan et al. [49] reported a complete cholangioscopy removal of biliary cast using single-operator cholangioscopy in a single sitting.

Biopsies samples of the stricture site under direct visualization with the use of SpyBite forceps have been done successfully after evaluation of mucosal abnormalities [50, 51]. Balderramo et al. [50] described 2 different cholangioscopic anastomotic stricture patterns, based on direct view. That may help to predict responses to endoscopic therapy. Pattern A was defined as mild erythema, and had better response to endoscopic treatment than pattern B characterized by edema, ulceration and sloughing. The histological findings showed nonspecific inflammatory changes.

## 9. Primary Sclerosing Cholangitis

The role of cholangioscopy in Primary Sclerosing Cholangitis (PSC) is to perform imaging of the biliary tract aiming at studying biliary strictures, characterizing dominant bile duct stenosis, enabling target biopsies of dysplastic lesions, and management of biliary stones.

Awadallah et al. [52] evaluated dominant strictures and cholangioscopy-directed stone therapy in PSC with demonstrable clinical benefits. Some other studies [53–57] have shown the effectiveness and usefulness of cholangioscopy in PSC, improving the detection of dysplastic lesions and allowing directed biopsies.

## 10. Resections

Although there are no published data on the therapeutic applications of cholangioscopy for the resection of a biliary lesion, a biliary polypoid lesion could be removed using a 5-F snare [14].

## 11. Conclusion

New therapeutic applications for cholangioscopy are emerging in the last years. Diffusion of single-operator concept, addition of digital imaging, and increase of availability of cholangioscopes surely played an important role.

The development of new accessories, as well as controlled trials evidence, will contribute in the near future to expand the indications of interventional cholangioscopy.

## Figures and Tables

**Table 1:** Cholangioscopy modalities.

Type		Advantages	Disadvantages	Endoscope diameter	Work channel
Dual-operator	“Mother-baby”	It was the first optical choledoscope developed	Necessary of two experienced endoscopists, low image quality, difficulty in handling, fragility, limited capacity of suction and irrigation, and small diameter of working channel, limiting therapeutic procedures	“Mother”: 12.6 mm “Baby”: 2.8–3.4 mm	0.8–1.2 mm
	Karl Storz (short-access-mother-baby)	More maneuverability, short size with less fragility, larger work channel	Necessity of two experienced endoscopists, only two-way deflected steering tip	“Mother”: 12.6 mm “Baby”: 3.4 mm	1.5 mm

Single-operator	Boston Scientific (SpyGlass)	Only one endoscopist, four-direction tip deflection	High cost, work channel diameter	3.3 mm	1.2 mm
	Ultra-slim endoscopes (direct peroral cholangioscopy) (Olympus, Pentax, Fujinon)	Superior video image quality with narrow band imaging capability, larger size of the work channel	High cost, can only be performed in dilated bile ducts, difficulty of insertion into the bile duct, lack of stability	5-6 mm	2.0–2.2 mm
